# 

**Published:** 1887-05-07

**Authors:** 


The Hospital.

An Institution, Family, and Parochial Journal of Hospitals, Asylums, and all Agencies for the Care of the Sick, Criticism and News.

SATURDAY, MAY 7TH, 1887.

### NOTICE TO ADVERTISERS.

*To insure prompt insertion all Advertisements for* THE HOSPITAL *must be sent in direct to the Advertising Agent, Mr. A. P. Watt, 2, Paternoster Square, E.C.*

### COVERS FOR BINDING

VOL. I. of THE HOSPITAL may be had of the Publishers, Messrs. WHITING & Co., Sardinia Street, Lincoln's Inn Fields, and of all Booksellers. Price 1*s.* 6*d.* each.

Country Subscribers are requested to order the Covers through their Booksellers, as they cannot be forwarded by post at book rate.

### THE LITERARY PRIZE.

A PRIZE of **Two Guineas** will be given every month for the best story or incident based upon hospital, or asylum, or student life, or any incident connected with the professions of medicine or nursing.

*** THE CHILDREN'S CORNER and AMUSEMENTS WITH PROFIT will be found on page vi of this week's issue.

### THE IN- AND OUT-PATIENTS' HOSPITAL DIARY.

This Diary is so arranged as to make some portion of it appear every week, and the whole in each monthly part. It has been very difficult to compile, and corrections will at all times be gratefully received. It has been suggested that similar information about the larger Provincial and Scotch Hospitals would be useful to many readers. We should be glad to hear if this is felt to be the case.

### I.—GENERAL HOSPITALS.

| Hospitals and Terms of Admission. | Physicians, Surgeons, and Medical Officers, with Days and Hours of Attendance. |
|---|---|
| Charing Cross Hospital, West Strand, W.C. Sec., Mr. A. E. Reade<br>Urgent cases are immediately admitted as in-patients. Subscriber's letter expected in other cases<br>Out-patients admitted without subscriber's letter first time; for continued attendance they must be obtained from the Secretary | *In-Physicians*, Drs. Pollock, M. Th.; Green, W. S.; Bruce, Tu. F. Hour, 1.30<br>*In-Surgeons*, Messrs. Barwell, M. Th.; Bellamy, Tu. F.; Bloxam, W. S. Hour, 2<br>*Out-Physicians*, Drs. Wilcocks, M.Th.; Abercrombie, Tu. F.; Lubbock, W. S.; Murray, W. S. Hour, 1.30<br>*Out-Surgeons*, Messrs. Cantlie, M.Th.; J. H. Morgan, Tu.F.; Stanley Boyd, W. S. Hour, 1.30 |
| "Dreadnought" Hospital (Seamen's Hospital Society), Greenwich, S.E. Sec., Mr. W. T. Evans<br>Entirely free to seamen and to all accident cases | *In- and Out-Physicians*, Drs. Curnow, Tu. Th. S.; H. T. Griffiths, M.W.F. Daily, between 9 and 3<br>*In- and Out-Surgeons*, Messrs. W. J. Smith, G. R. Turner. Daily, between 9 and 3 |
| French Hospital, 10, Leicester Place, Leicester Sq., W.C. Sec., Mr. E. Rimmel<br>Free to urgent cases and to all foreigners speaking French | *In-Physician*, Dr. Vintras, Tu. S. 10<br>*In-Surgeons*, Sir W. MacCormac, Tu. 9; Mr. J. Késer, W. F. 10<br>*Out-Physician*, Dr. Vintras, Tu. S. 10<br>*Out-Surgeons*, Messrs. H. de Méric, M. Th. 10; J. Késer, W. F. 10 |
| German Hospital, 113, Dalston Lane, E. Sec., Mr. C. Feldmann<br>Free to natives of Germany, and others speaking German, and to all accidents and emergencies. Others by Governors' letters | *In-Physicians*, Drs. Weber, M. T. 2; Port, Tu. F. 9 a.m.<br>*In-Surgeons*, Drs. Lichtenberg and Burger, W. S. 2<br>*Out-Physicians*, Drs. Tuchmann, Tu. W. 2; Ludwig, M. Th. 2; Keller, Tu. W. F. 2; Philippi, M. Th. F. 2 (*Men*, Tu. Th.; *Women*, M. W. F.) |
| ——— Eastern Branch, 49, Mansell Street, Aldgate, E. | *Out-Physician*, Dr. C. Harrer, Tu. F. 8 to 10 a.m. |
| ——— Western Branch, 259, Oxford Street, W. | *Out-Physician*, Dr. M. Castaneda, M. T. 8 to 10 a.m. |
| Great Northern Central Hospital, Caledonian Road, N. Sec., Mr. W. T. Grant<br>Free to all, without letters of recommendation | *In-Physicians*, Drs. Cholmeley, Cook, and Burnett, M. Tu. W. Th. F. 2<br>*In-Surgeons*, Messrs. W. Adams, W. Spencer Watson, and J. Macready, M. Tu. W. Th. F. 2<br>*Out-Physicians*, Drs. Beale, M. Th. 2; Beevor, Tu. 2, S. 10<br>*Out-Surgeons*, Messrs. Lockwood, M. Th. 2; Makins, Tu. F. 2 |
| Guy's Hospital, St. Thomas's Street, S.E. Supt., Dr. C. J. Steele<br>Patients are admitted, if beds are available, without letters of recommendation, on application at the Hospital. Out-patients are seen free without letters of recommendation | *In-Physicians*, Drs. Pavy, Tu. F.; Pye-Smith, M. Th. S.; Taylor, M. Tu. Th. F. Hour, 1.30<br>*In-Surgeons*, Messrs. Bryant, M. Th. Durham, M. Th. F.; Howse, W. S.; D. Colley, M. Th. Hour, 1.30<br>*Out-Physicians*, Drs. Carrington, W.; Hale White, M.; Goodhart, M. Tu. Th. F. Hour, 12.30<br>*Out-Surgeons*, Messrs. Lucas, Th.; Golding Bird, S.; Jacobson, W.; Symonds, M. Hour, 12.30 |
| King's College Hospital, Portugal Street, W.C. Sec., Mr. T. Mosse Macdonald<br>Accidents and urgent cases free at all times. Other cases by Governors' letters, but where not obtainable, application may be made to the Secretary | *In-Physicians*, Drs. Beale, Duffin, Yeo, Tu. W. F. 1.30; Tu. W. F. S. 2<br>*In-Surgeons*, Mr. Wood, Sir J. Lister, Messrs. H. Smith, W. Rose. Daily, 1.30<br>*Out-Physicians*, Drs. Ferrier, J. Curnow, N. I. C. Tirard. Daily. 1.<br>*Out-Surgeons*, Messrs. W. Rose, W. Cheyne, A. B. Barrow. Daily, 1. |
| London Hospital, Whitechapel Road, E. Sec., Mr. A. Haggard<br>Accidents and urgent cases free; out-patients for dental and maternity departments need no recommendation; other cases by subscriber's order. Out-patients are required to attend only once, unless otherwise directed by the Physician or Surgeon | *In-Physicians*, Drs. Mackenzie, Tu. F.; Down, Tu. F.; H. Jackson, M. Th.; Sutton, M. Th.; Fenwick, Tu. F. 1.30<br>*In-Surgeons*, Messrs. Rivington, Tu. F.; Couper, Waren Tay, M. Th.; McCarthy, W.S.; Treves, Tu.F. Hour, 1.30<br>*Out-Physicians*, Drs. Sansom, Th.; M. Ralfe, M. Th.; Turner, Tu. F.; Warner, Tu. F.; Gilbart Smith, W. S. J. Anderson, W. S. Hour, 1.30<br>*Out-Surgeons*, Messrs. Mansell-Moullin, M. Th.; Eve, M. W.; Reeves, Tu. S.; H. Fenwick, Tu.F. Hour, 1.30 |

| Hospitals and Terms of Admission. | Physicians, Surgeons, and Medical Officers, with Days and Hours of Attendance. |
|---|---|
| London Temperance Hospital, Hampstead Road, N.W.<br>For treatment without alcohol. By letter or small payment | *In- and Out-Physicians*, Drs. J. Edmunds, M.; J. J. Ridge, Tu. F. Hour, 1.30<br>*In- and Out-Surgeons*, Mr. A. P. Gould Th. 2.30 |
| Metropolitan Free Hospital, Kingsland Road, N. Sec., Mr. G. Croxton<br>Admission free to the sick poor, without any letter of recommendation. Urgent cases at all times | *In- and Out-Physicians*, Drs. Drysdale, Tu. F. 12; J. G. Dudley, W. S. 12; Fotherby, M. Th. 12; H. H. Tooth, Tu. F. 9; A. Haig, W. S. 9; A. Davies, M. Th. 9<br>*In- and Out-Surgeons*, Messrs. E. J. Chance, W. S. 9; Goodsall, Tu. F. 9; Walsham, M. Th. 9; A. A. Bowlby, F. 9; S. Paget, Th. 9; C. J. Thompson, daily, 9 |
| Middlesex Hospital, Berners Street, W. Sec. and Supt., Mr. A. O. D. Bartholeyns<br>Urgent cases admitted at all times; other cases by ticket. All medical cases must, in the first instance, be seen by the Resident Medical Officer, who attends at the hospital daily, at 11 a.m. | *In-Physicians*, Drs. Cayley, M. Th.; Coupland, Tu. F.; D. Powell, M.Th.; Finlay, Tu. F. Hour, 1.30<br>*In-Surgeons*, Messrs. Hulke, M. Th.; Lawson, M. Th.; Morris, Tu. F. Hour, 1.30<br>*Out-Physicians*, Drs. Fowler, Tu. F. 3.30; Biss, M. Th. 1.30; Pringle, W. S. 1.30<br>*Out-Surgeons*, Messrs. Andrew Clark, M. 1.15 (*Men*); Th. 1.15 (*Women*); Pearce Gould, F. 1.30 (*Men*); Tu. 1.30 (*Women*); J. B. Sutton, W. S. 9 |
| Miller Hospital and Kent Dispensary, Greenwich, S.E. Hon. Sec. Mr. W. Bristow. | *Physicians*, Drs. T. Creed, C. H. Hartt, G. Willes<br>*Surgeons*, Messrs. T. Moore, J. Poland, E. Clarke |
| North-West London Hospital, 18, Kentish Town Road, N.W. Sec., Mr. A. Craske<br>By subscribers' tickets or payment, but accidents and urgent cases free at all times | *In- and Out-Physicians*, Drs. W. C. Hood, J. Shaw, Edwarde H. Campbell. Daily, 1.30<br>*In- and Out-Surgeons*, Messrs. F. Durham, Collier, Jennings, J. Black. Daily, 1.30 |
| Ospedale Italiano, 41, Queen Square, Bloomsbury. Sec., Sig. L. Bonacina<br>Free to all, but Italians have preference | *In- and Out-Physicians*, Drs. M. Castaneda, Tu. F. 10 to 11; B. Sassella, M.Th. 10 to 11; Mr. J.W.B. Steggall, W.S. 4 to 5 |
| Poplar Hospital, 303, East India Dock Road, E. Sec., Lt.-Col. Feneran<br>Free to accidents and emergencies | *In-Surgeons*, Messrs. F. M. Corner, M. Brownfield, and Resident Surgeons<br>*Out-Surgeons*, Dr. J. D. Grant, W. S. Mr. T. E. Bowkett, Tu. F. 12 to 2 |
| Royal Free Hospital, Gray's Inn Road, W.C. Sec., Mr. J. S. Blyth<br>Admission free to the sick poor, without any letter of recommendation | *In- and Out-Physicians*, Drs. Cockle, M. Th.; Sainsbury, Tu. F.; West, W. S. Hour, 1<br>*In- and Out-Surgeons*, Messrs. Rose, M. Th.; W. Gant, Tu. F.; Barrow, W. S. Hour, 1 |
| St. Bartholomew's Hospital, Smithfield. Clerk, Mr. W. Cross<br>Admission free, without letter or ticket. Accidents and other urgent cases admitted at any time; ordinary cases of disease daily, between 9 and 10. Patients are seen for Electrical treatment on M. Tu. Th. and F. 1.30 | *In-Physicians*, Drs. Andrew, Church, Gee, Sir D. Duckworth. Daily, 1.30<br>*In-Surgeons*, Messrs. Savory, T. Smith, Willett, Langton, M. Baker. Daily, 1.30<br>*Out-Physicians*, Drs. Hensley, M. Th. 11; Brunton, Tu. F. 11; Legg, W. S. 11; N. Moore, Tu. W. Th. S. 9<br>*Out-Surgeons*, Messrs. Marsh, Tu. F.; 12; Butlin, M. Th. 12; Walsham, W. S. 12; Cripps and Bruce Clarke, daily, 9<br>*Casualty Physicians*, Drs. Davies, Tu. W. F. S.; O. Browne, M. Tu. Th. F.; Nias, M. W. Th. S. Hour, 9 |
| St. George's Hospital, Hyde Park Corner, S.W. Sec., Mr. C. L. Todd<br>Free to accidents and emergencies. Other in-cases by Governor's or subscriber's letter, but, in necessitous cases, this recommendation is not strictly insisted on. Letters are not required for out-patients. Vaccination on Th. at 11 | *In-Physicians*, Drs. Wadham, M. F.; Dickinson, M. Tu. Th. F.; Whipham, Tu.W. S.; Cavafy, Tu. S. Hour, 1<br>*In-Surgeons*, Messrs. Holmes, M. Th. F. 1, W. 1.30; Rouse, M. Th. F. 1, W. 1.30; Haward, Tu. Th. S. 1, W. 1.30<br>*Out-Physicians*, Drs. Ewart, M. F.; Owen, Tu. S. Hours, 10.30 to 12<br>*Out-Surgeons*, Messrs. Bennett, M. F.; Dent, Tu. S. Hours, 10.30 to 12 *Men*, F. S.; *Women*, M. Tu. |

### THE IN- AND OUT-PATIENTS' HOSPITAL DIARY.

### GENERAL HOSPITALS.—*continued.*

| Hospitals and Terms of Admission. | Physicians, Surgeons, and Medical Officers, with Days and Hours of Attendance. |
|---|---|
| **St. Mary's Hospital**, Cambridge Place, Paddington, W. Sec., Mr. Pietro Michelli<br>In-patients are admitted by letter of recommendation. Urgent cases and accidents free at all times. Out-patients require a letter. Electrical treatment Tu. and F. 2 | *In-Physicians*, Sir Edward Sieveking, Tu. F. 1.45; Drs. Broadbent, M. Th. 1.45; Cheadle, W. 1.45, S. 9.30<br>*In-Surgeons*, Messrs. Norton, M. Th. 1.45; Owen, Tu. F. 1.45; H. Page, W. S. 1.45<br>*Out-Physicians*, Drs. D. B. Lees, W. S.; S. Phillips, M. Th; R. Macguire, Tu. F. Hour, 1.30<br>*Out-Surgeons*, Messrs. W. Pye, M. Th.; A. J. Pepper, Tu. F.; A. Q. Silcock, W. S. Hour, 1.30 |
| **St. Thomas's Hospital**, Westminster Bridge Road, S.E. Steward, Mr. F. Walker<br>Accidents and emergencies free at all times. Other cases by Governor's letter, but, where unobtainable, application should be made to the Steward by letter, or personally between 10 and 4. Vaccination on W. at 11.30 | *In-Physicians*, Drs. Bristowe, Tu. F.; Stone, M. Th.; Ord, M. Th.; J. Harley, Tu. F.; Gervis, Tu. F. Hour, 2<br>*In-Surgeons*, Messrs. Sydney Jones, Tu. F.; Croft, M. Th.; Sir W. MacCormac, M. Th. Hour, 2<br>*Out-Physicians*, Drs. Payne, Tu. F.; Sharkey, M. Th.; Gulliver, W. S. Hour, 1.30<br>*Out-Surgeons*, Messrs. Clutton, Tu. F.; Anderson, W. S.; Pitts, M. Tu. Hour, 1.30 |
| **Tottenham Training Hospital**, Tottenham, N. Director, Dr. Laseron<br>Free. Accidents and emergencies admitted at all times | *In-Physician*. Dr. Rasch, Tu. F. 3 to 5<br>*In-Surgeons*, Drs. Lichtenberg, M. Th. 3 to 5; E. H. May, M. Th. 3 to 5<br>*Out-Surgeon*, Dr. Lloyd Smith, M. W. 11 to 2; *Men* only, W. 7 to 8 p.m. |
| **University College Hospital** (or North London), Gower Street, W.C. Sec., Mr. N. H. Nixon<br>Accidents and emergencies free. All other cases by Governor's letter of recommendation | *In-Physicians*, Drs. W. Fox, Tu. Th. 2, S. 9.30; Ringer, M. W. F. 1.30; Bastian, M. 2.30, W. 10, F. 2.30; F. Roberts, Tu. 1.15, Th. 1.30, S. 9.30<br>*In-Surgeons*, Messrs. B. Hill, Tu. 2.15, W. 2, F. 2.15; C. Heath, M. W. F. 2; M. Beck, Tu. Th. S. 2<br>*Out-Physicians*, Drs. Gowers, W. S.; Poore, Tu. F.; Barlow, M. Th. Hour, 1.30<br>*Out-Surgeons*, Messrs. Barker, M. Th.; Godlee, Tu. F.; Horsley, W. S. Hour, 1.30 |
| **West London Hospital**, Hammersmith Road, W. Sec. and Supt., Mr. R. J. Gilbert<br>Accidents and emergencies free at all times. Other cases require a letter of recommendation. New cases must attend at 1.30 | *In-Physicians*, Drs. G. Rogers, D. W. Hood, F. Drewitt<br>*In-Surgeons*, Messrs. C. Keetley, F. Edwards, W. B. Clarke<br>*Out-Physicians*, Drs. Herringham, M. Th.; Ball, Tu. F.; S. Taylor, W. S. Hour, 2<br>*Out-Surgeons*, Messrs. Ballance, Tu. F.; Weiss, M. Th.; Wainewright, W. S. Hour, 2 |
| **Westminster Hospital**, Broad Sanctuary, S.W. Sec., Mr. S. M. Quennell<br>Accidents and urgent cases free at all times. Other cases require a letter of recommendation | *In-Physicians*, Drs. Sturges, M. Th. S.; Allchin, M. Tu. F.; Donkin, Tu. W. S. Hour, 1.30<br>*In-Surgeons*, Messrs. Cowell, M. Tu. Th.; Davy, M. Tu. F.; Macnamara, M. W. S. Hour, 1.30<br>*Out-Physicians*, Drs. De H. Hall, M. Th.; A. H. Bennett, Tu. F.; Murrell, W. S. Hour, 1.30<br>*Out-Surgeons*, Messrs. Cooke, M. Th.; Bond, Tu. F.; Barrow, W. S. Hour, 1.30 |

### II.—SPECIAL HOSPITALS.

### CANCER.

| | |
|---|---|
| **Cancer Hospital**, Brompton, S.W. Sec., Mr. W. H. Hughes<br>Entirely free to both in- and out-patients | *In- and Out-Surgeons*, Drs. A. Marsden, W.; H. Snow, M.; F. A. Purcell, F.; Messrs. F. B. Jessett, W.; C. Stonham, Th.; C. Jennings, Tu.; W. H. Elam, S. Hour, 2 |
| **London Hospital**, Whitechapel Road, E. | Daily, 1.30. *See* General Hospitals |
| **Middlesex Hospital**, Berners Street, W. Admission free | *In-Surgeons*, Messrs. Hulke, Lawson, and Morris<br>*Out-Surgeon*, Mr. Morris, Th. 1.30 |
| **St. Saviour's Hospital**, Osnaburg Street, N.W. Sec., Mr. E. Poitiers<br>By Governors' letters | *In- and Out-Physician*, Dr. A. Kennedy, 10.30 and 4 daily |

### CHILDREN'S DISEASES.

| Hospitals and Terms of Admission. | Physicians, Surgeons, and Medical Officers, with Days and Hours of Attendance. |
|---|---|
| **Alexandra Hospital for Children**, 18, Queen Sq., W.C. Sec., Mrs. Marsh<br>For hip disease | *Physician*, Dr. Steavenson<br>*Surgeons*, Messrs. H. Marsh Tu. F.; A. Bowlby, M. Th. Hour, 4.30<br>(No out-patients) |
| **All Saints' Children's Hospital**, 4, Margaret Street, W.<br>For chronic joint diseases | *Surgeon*, Mr. N. Smith<br>(No out-patients) |
| **Belgrave Hosp. for Children**, 77 & 79, Gloucester Street, Pimlico. Hon. Secs., Capt. Stopford, and Rev. J. Storrs<br>By letters; accidents free | *In- and Out-Physicians*, Drs. W. Hope and W. Ewart, M. Th. 9<br>*In- and Out-Surgeons*, Messrs. C. T. Dent and Margerison, Tu. F. 9 |
| **Charing Cross Hospital**, West Strand, W.C. | *Out-Physician*, Dr. Nursby, W. S. 1.30 |
| **Cheyne Hospital for Sick and Incurable Children**, 46, Cheyne Walk, Chelsea, S.W. Sec., Miss D. Evans | *Physician*, Dr. G. V. Poore<br>*Surgeons*, Messrs. T. P. Bartlett and J. Macready attend alternate days in afternoons. (No out-patients) |
| **East London Hospital for Children and Dispensary for Women**, Glamis Road, Shadwell, E. Sec., Mr. Ashton Warner<br>By Governor's letter. Urgent cases and accidents are admitted free | *In-Physicians*, Drs. Donkin, M. Th.; Eustace Smith, T. F.; Crocker, F.<br>*In-Surgeons*, Messrs. A. Cæsar, R. W. Parker, W. F.<br>*Out-Physicians*, Drs. Crocker, M.; Dawson Williams, Tu. Th.; Coutts, W. F. Hours, 1 and 3<br>*Out-Surgeon*, Mr. Dunn, Tu. F. 1, 3 |
| **Evelina Hospital for Sick Children**, Southwark Bridge Road, S.E. Sec., Mr. T. S. Chapman.<br>Free, but Governor's letter takes precedence. In-patients (boys) between 2 and 10, (girls) between 2 and 12 | *In-Physicians*, Drs. F. Taylor and J. Goodhart<br>*In-Surgeons*, Messrs. H. G. Howse and R. C. Lucas<br>*Out-Physicians*, Drs. N. Tirard, M. Th. 9; F. Willcocks, Tu. F. 9<br>*Out-Surgeons*, Messrs. C. J. Symonds, S. 9; G. H. Makins, W. 9 |
| **German Hospital**, Dalston Lane, Dalston, E. | *In-Physician*, Dr. Rasch, M. Th. 9 |
| **Great Northern Central Hospital**, Caledonian Rd. N. | *Physicians*, Drs. Murray and Barnes, W. 2.30 |
| **Grosvenor Hospital for Women and Children**, Vincent Sq., S.W. Hon. Secs., Mr. A. J. Ram and Miss Day<br>By letter or payment | *In- and Out-Physicians*, Drs. R. Gibbons and Steavenson, Tu. W. Th. S. 2<br>*In- and Out-Surgeons*, Messrs. Smythe and Parker, Tu. W. Th. S. 2 |
| **Hospital for Sick Children**, 48 and 49, Great Ormond St., W.C. Sec., Mr. S. Whitford<br>By Governors' letters. If without letter, the case of the applicant is investigated<br>In-patients between 2 and 12; Out-patients under 12 | *In-Physicians*, Drs. W. Cheadle, Tu. F.; Sturges, W. S.; Barlow, M. Th. Hours, 9 to 11<br>*In-Surgeons*, Messrs. H. Marsh, W. S.; E. Owen, W. S. Hours, 9 to 11<br>*Out-Physicians*, Drs. R. J. Lee and Abercrombie, M. Th.; Lubbock and Money, Tu. F. Hours, 8.30 to 10<br>*Out-Surgeons*, Messrs. Morgan and Pitts, W. S. 8.30 to 10 |
| **King's College Hospital**, Portugal Street, W.C. | *In- and Out-Physician*, Dr. T. C. Hayes, W. F. 1 |
| **Middlesex Hospital**, Berners Street, W.<br>Free. Children under 3 | *Out-Physician*, Dr. Duncan, W. S. 1.30 |
| **North Eastern Hospital for Children**, Hackney Road, E. Sec., Mr. A. Nixon<br>By free ticket or small payment | *In- and Out-Physicians*, Drs. W. Cayley, F. Turner, C. Semple, and W. Pasteur, M. W. Th. S. 1.30<br>*In- and Out-Surgeons*, Messrs. Waren Tay and Pollard, M. 1.30, F. 9 |
| **Paddington Green Children's Hosp.**, Paddington Green. Sec., Mr. W. H. Pearce<br>Admission free; boys under 12; girls under 14 | *In- and Out-Physicians*, Drs. T. Lauder Brunton, Ogilvie, Tu. F.; G. Laycock, M. Th.; S. Phillips, W. S. Hour, 9.15<br>*In- and Out-Surgeons*, Messrs. W. W. Cheyne, M., S. Boyd, F. Hour, 9.15 |
| **Royal Hospital for Children and Women**, Waterloo Bridge Road, S.E. Sec., Mr. R. G. Kestin<br>Out-Patients pay 1*d.* on each attendance. Boys not admissible, as out-patients, over 12, or, as in-patients, over 8 | *In-Physicians*, Drs. Park, M. Th.; Gulliver, Tu. F.; Price, W. S. Hour, 2<br>*In-Surgeon*, Mr. W. H. A. Jacobson, M. Th. Hour, 2<br>*Out-Physicians*, Drs. Park, M. Th.; Dakin, W. F.; Hadden, Tu. S. Hour, 2<br>*Out-Surgeon*, Mr. E. O. Day, W. 2 |
| **St. Mary's Hospital**, Cambridge Place, Paddington | *Out-Physician*, Dr. M. Handfield Jones, M. Th. 1.30 |
| **St. Thomas's Hospital**, Westminster Bridge Road, S.E | *Out-Physician*, Dr. Cory, W. S. 1.30 |

### THE CHILDREN'S CORNER.

**Third Quarterly Prize Competition.**
Began 2nd Apr., 1887 ; ends 25th June, 1887.

**Puzzles—Sixth Week.**

No. 24. BURIED NAMES OF BIRDS. A letter of the alphabet; a word meaning jet-black; a place where goods are bartered and a preposition ; a kind of fun.

No. 25. CHARADE.

In my *first* for ever flow
Sounds of joy and sounds of woe ;
In my *second*, newly made,
Thousands ev'ry year are laid ;
In my *whole* we never jest,
Prayers are made, and sins confess'd.

No. 26. CONUNDRUM. What is that which is often weighed, but never with the intention of taking its weight.

| | | | |
|---|---|---|---|
| 1 | 2 | 3 | 4 |
| 5 | 6 | 7 | 8 |
| 9 | 10 | 11 | 12 |
| 13 | 14 | 15 | 16 |

No. 27. SQUARE PUZZLE. A square is marked out into 16 parts, as in the sketch. A boy stands in No. 1, and is offered a reward if he can find out how to get to No. 16. The conditions are these: he must pass through *every* small square; he must enter each square *only once*; and he must go laterally, *i.e.*, from 1 to 5 or 1 to 2, and so on; not from 1 to 6, etc. How did he do it?

**Letters—Sixth Week.**

The Letter-subject for next week will be "Music."

**Results of Fourth Week—Puzzles.**

No. 15. DECAPITATION. Cod.

No. 16. BIRDS' NAMES ENIGMATICALLY EXPRESSED. Swallow, Nightingale, Wren, Starling, Skylark.

No. 17. ENIGMA. Chocolate.

No. 18. BURIED WORDS. Hospital.

No. 19. CONUNDRUM. Why has Mr. Gladstone never been able to insure his life? Because no one could ever be found who could make out his policy. (I fear this is a Conservative riddle.)

*All right*—R. Epton, Gom, Umslopogaas, Alsie, Tim, Skye, Nordisa, Sutton, Joe; *Four right*—Scrooge, A. C. K., A. G. S. McCulloch, Peeler, Mikado, Ada, Dot, Bohemian Girl, The Eda; *Three right*—Ellie.

(Ellie is credited with three right, and Sutton with two right, in last week's answers.)

**Letters.**

Flowers are nature's jewels. They are among the very first objects which enchain the attention of the young, and their pure and ennobling influence clings to us through life. Children and flowers! Can we wonder at the union of these terms when we see how much they have in common—their sweetness, their innocence, their purity? Lovely flowers, says Wilberforce, are the smiles of God's goodness. They wreathe the cradle, the altar, and the tomb, while their fragrance and their beauty make them treasured adornments of the sick-room. The letters which I have received from my little friends all breathe the same spirit—an ardent love for these beautiful emblems of life. Mikado glories in the beauties of the rose—the Gloire de Dijon or the Marshal Niel; the only rose she does not love is the last rose of summer! Skye thinks the spring flowers are far fresher and sweeter than any other, and she gossips delightfully about violets, primroses, lilies, cowslips, and forget-me-nots.

A new little friend, who has chosen the pretty *nom de plume* of Sunshine, writes me:—

DEAR MR. EDITOR,—I have a small garden, and some very nice flowers. There are some woods only about half an hour's walk from here. There are dog-violets, primroses, and anemones there. I am very fond of flowers. I went to a flower-service last summer, and took a bunch of flowers, and a little boy, my friend, took some sunflowers. I liked the service very much.—I am, yours truly, SUNSHINE.

Lynden Lodge, Elmfield Road, Bromley.

Ada speaks of the universality of the love of flowers, their usefulness, apart from their beauty, and the good fruit which may result from their culture in withdrawing the poor man, who cultivates them, from indulgence in drink and evil company. Ada's letter is at once sensible and practical, and a great credit to a little girl who is only 12. Ellie tells me that roses and violets are her favourites, and she adds that she is just now learning that beautiful poem on the "Rose" by Dr. Watts, commencing:

"How fair is the rose, what a beautiful flower,
The glory of April and May.
But its leaves are beginning to fade in an hour,
And they wither and die in a day."

Here is a note from Bohemian Girl:—

DEAR MR. EDITOR,—I am very fond of flowers. I think lilies of the valley and forget-me-nots are my favourites. I was in London on "Primrose Day," and saw the Beaconsfield Statue with all its wreaths; one beautiful one had the words "At Rest" in violets amongst the primroses. The wild flowers in our neighbourhood are very beautiful, some of the hedges are full of wild roses and honeysuckle.

Yours truly, BOHEMIAN GIRL.

12, Upper Tichborne Street, Leicester.

Peeler, in the course of a most interesting letter about the wonderful variety of flowers one sees in country hedgerows, observes, "When looking at a bunch of fresh-gathered flowers, my thoughts turn to a hospital ward in London, with its little white beds arranged round the room, and sickly, childish faces peeping out of the coverlets. A lady enters the ward with a bunch of cowslips, which she places in a glass in the middle of the ward where all can see. Oh! how those faces light up with pleasure at the sight so common to us country folk, but how dear to all Londoners. Will not some of our readers, now that the primroses and cowslips are coming out, remember the hospitals in London, and how gratefully received would even a small bunch be? It would cheer many a poor little suffering one, to gaze and dream of the country."

One mark each:—Bohemian Girl, Ellie, Mikado, Peeler, Skye, Ada, Sunshine.

(One mark each is also credited to Peeler and Ethel for previous week.)

Answers must be addressed to the "PUZZLE EDITOR," THE HOSPITAL, Norfolk House, Norfolk Street, Strand, W.C., not later than Thursday next. The "Children's Corner" Coupon (see advertisement pages) must be enclosed.

### AMUSEMENTS WITH PROFIT.

**No. 5. Double Acrostic.**

My primals and finals make up a phrase,
That now I'm quite sure is meeting your gaze.
* * * * * * *
In the eighth Harry's reign this female martyr died ;
A Florentine by whom *Del Principe* was writ ;
'Tis a town in Holland—the velvets are fam'd wide ;
A jocular divine—his *Sermons* show his wit ;
This is a thoroughfare within the City found ;
A dramatist—one play his memory keeps green ;
A nymph of old ; a paper ; a peculiar sound ;
A river port of Cherson, on Russia's map is seen ;
A town near Rome, for its glorious ruins known ;
A "prophetess" whom yet a few believers own!

**No. 6. Mathematical Question.**

A person sells a horse for £144, and gains as much per cent. as the horse cost him. What was the price of the horse?

**Answers.**

No. 1. DOUBLE ACROSTIC.

O b I
S ura T
P atagoni A
E rriga L
D elh I
A ndorr A
L eyde N
E br O

Correct answers have been received from Robin, Ostrich, Canice, Letheador, K. M., Broadwater, Aralc, Mater, Gretta, A. M. E., May, Dawnlover, Ellice, The Man of the Sea, Gip, Perseverance.

(We are aware that "hospital" is written both "ospedale" and "ospitale" in Italian, and that likewise there is a form "spedale," answering to our "spital." However, the title of the Italian Hospital in London (as may be seen from our *Diary*) is invariably written as we give the solution.)

No. 2. MATHEMATICAL QUESTION.

The mouse advances one foot in a day and a night. On the seventeenth night, therefore, it has advanced seventeen feet; and, as it goes up three feet every *day* (dropping two during the night), on the eighteenth day it will reach the top.

Answer, 18 days.

Correct answers have been received from Letheador, Perseverance, Gip, The Man of the Sea, May, A. M. E., Gretta, Hexameter, Mater, Broadwater, K. M., Canice, Ostrich, Robin, Ellice.

"HELP GUY'S."—The following competitors have practically answered A. M. E.'s suggestion:—Gip and two friends, 3s.; K. M., 1s. Three or four others have promised to do so. We shall be happy to forward to the Treasurer of Guy's any sums our friends may send us. A. M. E. now promises 2s. 6d. if £1 is sent in by the solvers.

Answers must be addressed to THE PRIZE EDITOR, AMUSEMENTS WITH PROFIT, THE HOSPITAL, Norfolk House, Norfolk Street, Strand, W.C., not later than the *first* post on Saturday next. The full name and address must in all cases be given, but a *nom de plume* may be added for publication. Only one side of the paper should be written on. The "Amusements with Profit" Coupon (see advertisement pages) must be enclosed with replies.

